# The origin of the Acheulean. Techno-functional study of the FLK W lithic record (Olduvai, Tanzania)

**DOI:** 10.1371/journal.pone.0179212

**Published:** 2017-08-02

**Authors:** Policarpo Sánchez-Yustos, Fernando Diez-Martín, Manuel Domínguez-Rodrigo, Javier Duque, Cristina Fraile, Isabel Díaz, Sara de Francisco, Enrique Baquedano, Audax Mabulla

**Affiliations:** 1 Department of Prehistory and Archaeology, University of Valladolid, Pza. del Campus, Valladolid, Spain; 2 Department of Prehistory, Complutense University, Madrid, Spain; 3 IDEA (Instituto de Evolución en África), Museo de los Orígenes, Madrid, Spain; 4 Museo Arqueológico Regional, Alcalá de Henares, Madrid, Spain; 5 Archaeology Unit, University of Dar es Salaam, Dar es Salaam, Tanzania; Max Planck Institute for the Science of Human History, GERMANY

## Abstract

The Acheulean materials documented in FLK West dated c. 1.7 Ma. are the focus of the present work. An original techno-functional approach is applied here to analyze the origin of Acheulean tools. According to the results, these tools were employed in different functional contexts in which tasks of different durations that transformed resources with different resistances were carried out. The exploitation of large and resistant resources suggests that the economic mechanism governing the manufacture of these tools was an increase in the demand of the work load. The decision processes underlying the production of these tools have thus an evident functional motivation. However, the presence of a refined handaxe in the studied sample indicates that the design form and production principles of handaxe manufacture were the result of an abrupt emergence rather than a long gradual development. The integration of mechanical and ergonomic investigation in our research has been crucial to explain how a core-and-flake industry gave way to a technology based on the production of large and heavy shaped tools.

## Introduction

The earliest known evidence of the Acheulean technology dates back to c. 1.7 Ma. andis limited to three sites: KS4 (West Turkana, Kenya), KGA6-A1 (Konso, Ethiopia) and FLK West (Olduvai Gorge, Tanzania) [1–3]. The regional distribution of these sites suggests a rapid dispersal of Acheulean technology across Eastern Africa. Among these sites, FLK W constitutes the chronologically best-braked site, as the dates obtained (1.69 and 1.66 Ma) from tuffaceous sediments underlying and overlying the site are close in time, and the Acheulean materials are stratigraphically situated much closer to the lower tuff [3]. The chronology of KS4 and KGA6-A1 was estimated in 1.76 Ma due to a complex age model largely based on interbasin correlations (Konso and Turkana) [1–2]. However, this dating procedure suggests an open margin for chronological variation [3]. Despite the uncertain chronological differences that may exist among these three lithic assemblages, to date, they represent the earliest Acheulean evidence (c. 1.7 Ma) due to their chronology and the strong techno-typological affinities they show [1–3].

The available information about these remarkable sites is still insufficient to establish a comprehensive framework of the earliest Acheulean phenomenon. However, it is possible to identify some common techno-typological attributes among the lithic assemblages of these three sites, namely: 1) occurrence of Large Cutting Tools (LCTs) made on different rocks and blanks; 2) the crudeness of the LCTs shaping hinders their typological classification, but picks, handaxes and cleavers can be recognized; 3) shaping is mainly focused on edge modification and is hardly ever aimed at managing the whole volume of the piece; 4) the management of bifacial and/or bilateral planes is very rare; 5) and planform symmetry is virtually absent [1–4].

The number of Acheulean sites in Africa increases notably between 1.6 and 1.3 Ma [5–12]. Indeed, during this period the first Acheulean assemblages outside Africa are recorded [13–14]. Despite the increasing amount of available data and even the application of new approaches (i.e. technological), there are still two critical questions left to be resolved: 1) How and why did the Oldowan give way to the Acheulean? 2) Did the Acheulean evolve during the early Pleistocene in Africa (>0.78 Ma)?

One of the central elements in current debates about human prehistory and evolution is the transition from the Oldowan to the Acheulean [15], since it is the critical step when hominins moved from an *ad hoc* technology to one where the structure of the tool required greater preparation and planning. However, the cultural processes that led to the Acheulean emergence are poorly understood. In recent years, some authors have suggested epistemological and methodological issues as the reasons that may explain this difficulty [7, 16–19]. More specifically, these authors claimed that the classical definition of the Acheulean, based on culture history postulates from which the typological tradition bloomed, possesses important ambiguities that have fueled the long standing Developed Oldowan/Acheulean debate and, in turn, may explain why it is still unclear how and why the Oldowan gave way to the Acheulean. It should be pointed out that the archaeological identity of the Acheulean is rooted in a simplistic nineteenth-century typological postulate by which the occurrence of handaxes and other LCTs indicates the Acheulean character of any given assemblage. In fact, the presence of handaxes and other LCTs is considered nowadays the main proxy of the Acheulean emergence [1–3].

On the other hand, current studies [2, 5, 20] centered on the sequences of Olduvai (Tanzania), Konso (Ethiopia) and Melka Kunture (Ethiopia) have re-edited the long running hypothesis which suggest that there was an evolution in the Acheulean during the early Pleistocene in Africa [21]. According to these studies, before 1.3–1.2 Ma the shaping processes are characterized by their crudeness in these sequences, while between 1.3–1.2 and 0.78 Ma handaxes become more refined (from edge modification to volume modification) and more symmetrical (symmetry in both plan view and cross-section). Such refinement resulted in larger flake scar count, a progressive three-dimensional symmetry, and standardization in both manufacturing and planform. Furthermore, diachronic variations in raw material procurement strategies and LCT operation sequencing are observed. This evolutive hypothesis, in turn, involves the assumption that while the origin of the Acheulean was rather abrupt, handaxe design forms were developed gradually, becoming more elaborate and sophisticated as time went by [2, 5, 21]. However, other archaeologists have not identified any evolutive pattern in the Olduvai sequence [22–24]. The scarcity of Acheulean assemblages and the oddity of massive and systematic production of handaxes and other LCTs between 1.3 and 0.78 Ma hinder an accurate understanding of how continuous or discontinuous was the trend towards refinement was within the Early Pleistocene Acheulean assemblages in East Africa [19]. The hypothetical evolution within the Acheulean on a global scale is also a current matter of discussion [25–26].

The main objective of the present paper is to shed light on the origin of the Acheulean tools, in order to shed light on how and why the Oldowan gave way to the Acheulean. To do this, we employ an original methodological approach to analyze a rich and very well contextualized early Acheulean record. Due to the particularity of the studied sample, we can also provide new elements to discuss in conceptual and technological terms whether handaxes could be the result of a gradual development. It is important to note that although we provide here new elements to shed light on the early Acheulean development, this issue should be addressed diachronically by comparing different assemblages [2, 5, 20]. Consequently, in a second stage, the heuristics elements introduced here might be applied to a broader scale.

## Materials and method

All necessary permits were obtained for the described study (Tanzania Commission for Science and Technology). The specimens involved in the study are publicly deposited in the Research station of Olduvai (Olduvai Gorge, Ngorongoro Conservation Area, Arusha, Tanzania). All relevant data are within the paper and its Supporting Information files.

**Fig 1:**
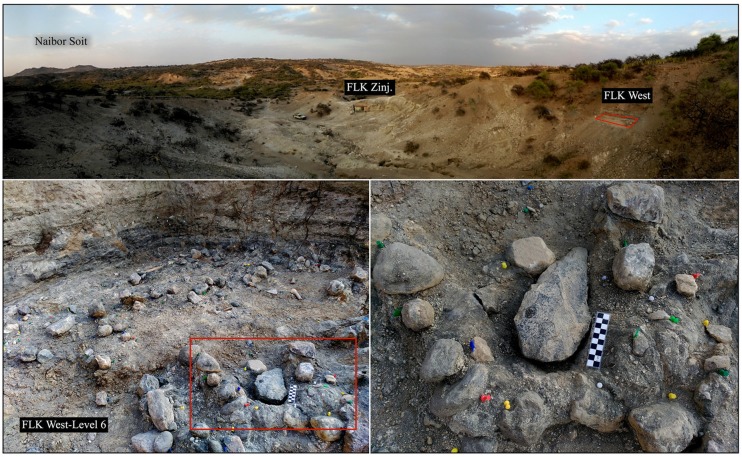
FLK W earliest acheulean site. Location and excavation details of FLK W Level 6.

The Acheulean materials documented in FLK West (FLK W, Lowermost Bed II, Olduvai) dated c. 1.7 Ma. are the focus of the present study [3]. The archaeo-stratigraphic sequence at FLK W is divided into 6 levels, and the Acheulean materials are concentrated in the lower levels. The 84 artifacts studied here are large flakes (≥10 cm) and complete LCTs, and came from the 2012–2015 field-seasons. Almost all of them were recovered in level 6 (n = 79) and a few in level 5 (n = 5) (Fig 1). Formally classified specimens and normative morphotypes (i.e. handaxe, pick and cleaver) are included within the category of Large Cutting Tools (LCTs), as well as other shaped large blanks (≥10 cm).

**Fig 2:**
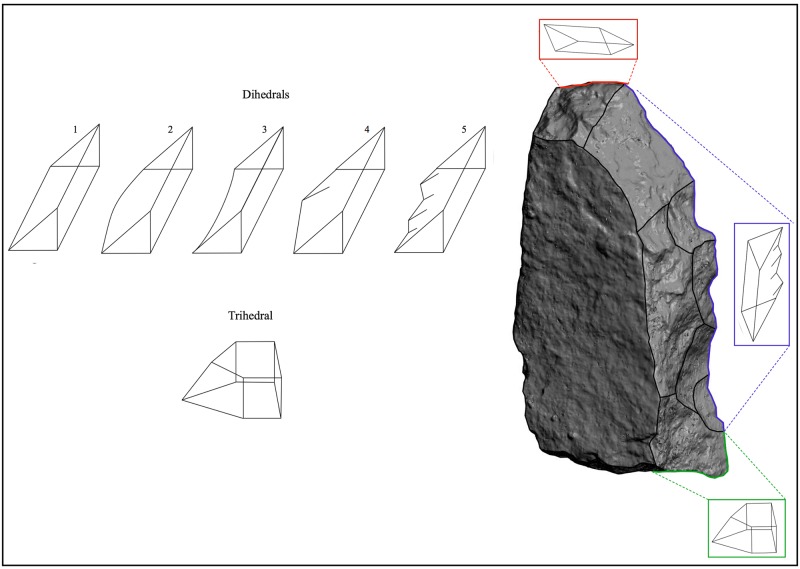
Working edge. (A) Geometry of working edge: Dihedral (edge with two planes) and Trihedral (edge with three planes). (B) Dihedrals delineations: 1) straight (not curved, flexed or scalloped line); 2) convex (curved outwards line); 3) concave (curved inwards line); 4) pointed (angular flexed line); 5) irregular (scalloped line).

We have adopted a techno-functional approach aimed at analyzing the origin of the Acheulean tools on the basis of the interplay between their design and functional capabilities (i.e. functioning). Tool design is addressed here by studying how the tool is manufactured, paying special attention on its shape, size and the attributes of the working edges. We consider a working edge as the junction of different planes (knapped or not) that form a dihedral or trihedral angle <90° that could be actioned. The transformative capacity of a working edge is determined by the edge form and the size and shape of the tool (volumetric structure) which, in turn, relates to the degree of loading force that may be applied [27–30]. The variables employed here to analyze the working edge form are: geometry (dihedral or trihedral), type of shaping (unifacial, bifacial or trifacial), length (measured by placing a sewing meter along the working edge), angle (along the working edge three readings were taken at 25%, 50% and 75% intervals using a digital clinometer and the mean value was recorded), and delineation (straight, irregular, pointed, convex and concave) (Fig 2). The studied artifacts have been sorted into three “tool-types” according to the geometry of their morphopotential structures: Type 1 (Tp1), artifacts with trihedral/s; Type 2 (Tp2), artifacts with trihedral/s and dihedral/s; and Type 3 (Tp3), artifacts with dihedral/s.

**Fig 3:**
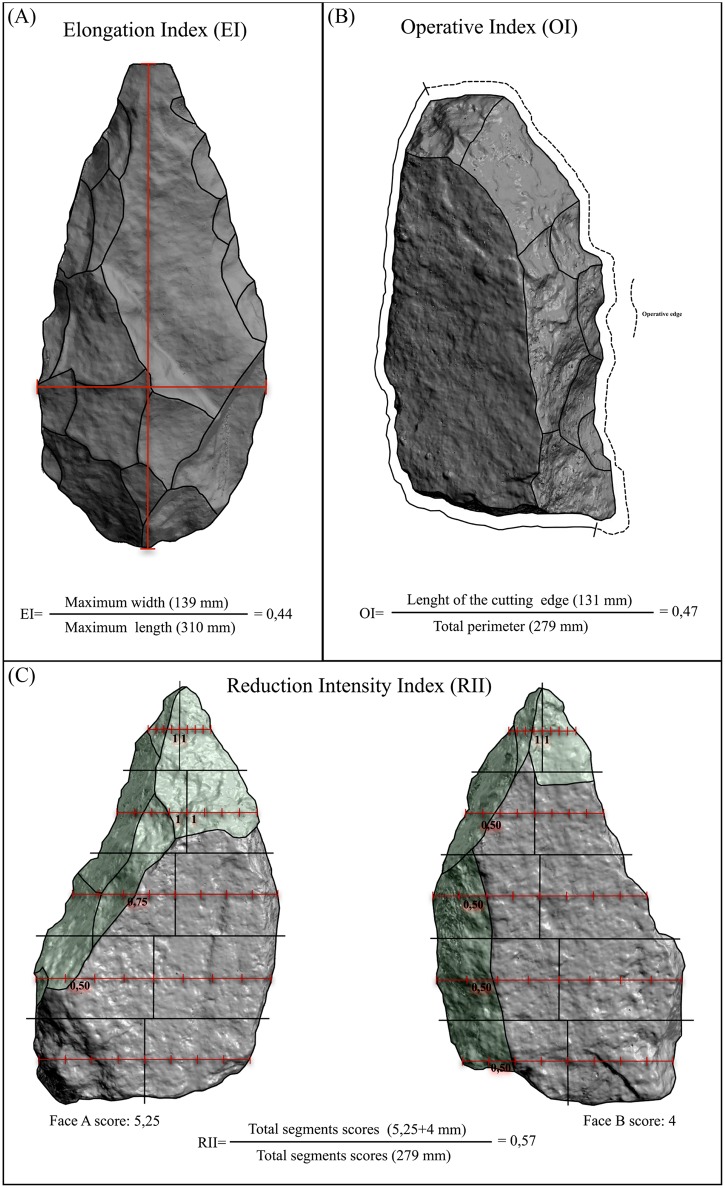
Shape and technological indexes. (A) Elongation Index. (B) Operative Index. (C) Reduction Intensity Index.

The other element in the tool design analysis refers to how the tool was manufactured and the working edges integrated within the overall volumetric structure. With this purpose we have employed four proxies: 1) Elongation Index (EI, maximum width divided by maximum length), recurrently employed to study “elongation” in Acheulean assemblages (Fig 3A), understanding elongation as a factor relating to the construction or use of a long axis [31]; 2) Operative Index (OI, length of working edges divided by the total perimeter of the artifact), applied to differentiate which part of the perimeter of the artifact (knapped or not) could be destined for transformative tasks (Fig 3B); 3) the Scar Density Index (the ratio of flake scar number to surface area), as a measure of handaxe reduction that was designed to test statistically whether or not handaxes were resharpened [32–33]; 4) Reduction Intensity Index (RII), applied to quantify the extent to which scars cover the surface area of the studied artifact (Fig 3C).

The RII is broadly similar to the Invasiveness Index [34] and the Flaked Area Index [35] since they share the same principle (scar coverage or flaked area increases with reduction) and procedure (segmentation of a handaxe into proportional sectors for accurate measuring of the flaked area). The procedure of the RII is to sub-divide each surface (shaped or not) of the tool into four analytical segments (distal, right lateral, proximal and left lateral), to sub-divide each segment into four zones (outer zone, middle-outer zone, middle-inner zone and an inner zone), and to ascribe an invasiveness score to each zone (0.25, 0.5, 0.75 and 1). Scores are attributed on the basis of the encroachment of flake scars in the middle of each segment (Fig 3C). The studied LCTs were scanned using a NextEngine 3D laser scanner, and 3D visualization software (3D Afanche) was employed to sub-divide the pieces into segments and zones and ascribe each zone to an invasiveness score. The scores of all zones are summed to give a total figure for the invasiveness of each artifact. Dividing this total by the number of segments (i.e. 16 for artifacts with two planes and 24 for three planes), gives a result ranging between ‘0’ (non-modified) and ‘1’ (completely modified) (Fig 3C). We sorted the artifacts into four types of reduction intensity using the RII in order to occasionally synthesize and compare information easily: RI 0, non-reduced artifacts (RII = 0.00); RI 1, slightly reduced artifacts (RII = < 0.25); RI 2, moderately reduced artifacts (RII = ≥ 0.25 – < 0.50); RI3, extensively reduced artifacts (RII = ≥ 0.50–1).

Within our tool design analysis we developed a simple method to observe *de visu* the frontal symmetry of handaxes. These are the steps followed: 1) divide the tool horizontally into 5 equal segments; 2) divide each segment horizontally and vertically into equal portions; 3) a red line is placed vertically in the point where the two lines that divide each segment intersect, its length represents the length of the segment; 4) and the more aligned the red lines are the greater the symmetry of the handaxe. Measurements were taken using the above-mentioned 3D visualization software.

Tool design determines the patterns of use and action of the tool (i.e. tool functioning). In other words, “la forme structure l'action” (see page 8 in [36]. The volumetric structure of the artifact implies a set of possible uses and gestures, while limiting others. The tool functioning analysis developed here is supported on the grounds of engineering research in the mechanics of cutting [37–42]. This research, applied to modern industrial interests, has identified the following four principles that guide cutting mechanics, but minor variations may occur depending on the tasks performed: 1) there is a positive correlation between edge angle and the loading force required to initiate a cut; 2) there is a positive correlation between scalloped or serrated edges and edge longevity; 3) there is a negative correlation between edge length and the time and number of cutting strokes required to cut a given portion of material; 4) and there is a positive correlation between tool mass and loading force and cutting stress. In short, cutting efficiency depends on the amount of energy expended to initiate a cut and the tool form attributes. Current investigation in the field of Paleolithic archaeology has brilliantly integrated mechanical and ergonomic research within functional and morphological analysis of lithic cutting technology through numerous experiments [28–30, 43–50].

**Fig 4:**
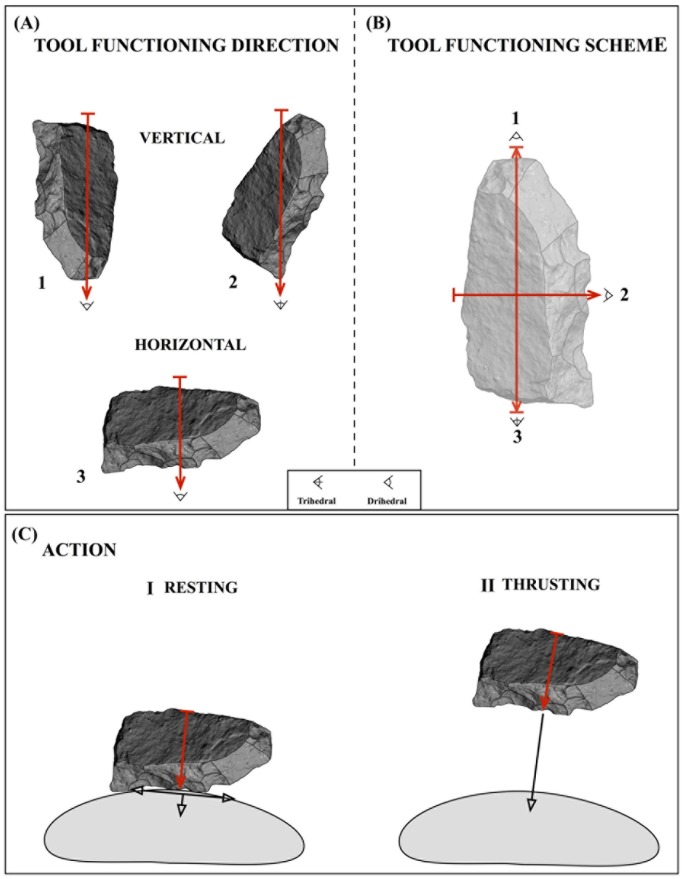
Tool functioning. (A) Tool functioning direction (TFD): 1. TFD: Vertical; Functional axis: Length axis (red arrow); Working edge location: Distal; 2. TFD: Vertical; Functional axis: Length axis (red arrow); Working edge location: Proximal; 3. TFD: Horizontal; Functional axis: Width axis (red arrow); Working edge location: Lateral. (B) Tool functioning scheme: Functioning axis direction (red arrows), working edge location (proximal: 1; distal: 2; lateral: 3), and geometry (dihedral: 1/3; trihedral: 2. (C) Action: resting (I) and thrusting (II) (black arrows indicate the direction of the push and pull forces exerted on the matter to transform), note that both actions are performed along the width axis/horizontal TFD (red arrow).

The behavior involved in tool design is much easier to infer than tool functioning [36]. To overcome this difficulty, we have developed a methodological strategy to analyze tool functioning based on the mechanical principles of cutting and focused on how the tool-user may transmit the force to cut, split or deform material. The two proxies employed are: tool functioning direction and tool action. Tool functioning direction (TFD) is designated by two parameters: the axis along which the force is transmitted (length axis or width axis) and the location of the working edge (distal, proximal, right lateral and left lateral). According to this, we distinguish two types of TFD: vertical TFD, the working edge/s is/are located in the distal and/or proximal region (single or double vertical TFD) and the force is transmitted along the length axis (Fig 4A.1 and 4A.2); and horizontal TFD, the working edge/s is/are located in the distal and/or proximal region (single or double horizontal TFD) and the force is transmitted along the width axis (Fig 4A.3). In overall terms, artifacts may indicate a vertical TFD (simple or double), a horizontal TFD (simple or double), or both TFD combined (vertical and horizontal). The TFD of an artifact is graphically represented in what we call the “tool functioning scheme”, where information about the functioning axis and working edge location (proximal, distal or lateral) and geometry (dihedral or trihedral) is codified (Fig 4B). It is important to note that TFD does not imply any action/motion (see Fig 4C).

On the other hand, we have been inspired by Leroi-Gourhan’s work [51] to scrutinize the set of possible actions that tools would allow a tool-user to make. Action refers to the cutting behavior that the tool-user employs. We distinguish between two theoretical actions: 1) “resting”, by this action the working edge rests on the matter to transform and the push and pull forces are exerted in a parallel and perpendicular direction respect to the plane to transform (Fig 4C.I). These forces can be modulated by wrist, hand and forearm motions. This action is suitable for tasks (e.g. slicing, scraping or sawing) that require precise cutting with accurate working edge placement; 2) “thrusting”, by this action force is applied by perpendicular or oblique downwards blows (Fig 4C.II). In this case, the pushing force largely depends on the momentum resulting from the tool mass and can be modulated by forearm and shoulder motions and body mass. This action is very convenient for heavy-duty tasks (e.g. chopping, cleaving or digging) that require the application of stronger forces through vigorous movements. According to the principle of cutting mechanics by which there is a positive correlation between the tool mass and the loading force and cutting stress, and due to the fact action refers to the way in which force is transmitted to the object to transform, we will use tool mass as the main proxy to infer tool action. The following assumption can be inferred from the cutting mechanics and behavior linked to the actions defined here: the lighter tools would be more efficient in resting actions than heavier ones, whereas heavier tools would be more efficient in thrusting actions than the lighter ones. In sum, in order to infer the theoretical action that the studied tools could have performed, we will try to identify the mass threshold below or above which the cutting efficiency may decrease significantly and a change of action would be required. Tools around that threshold may perform both actions without cutting efficiency decay.

## Results

### Tool design

A total of 84 artifacts have been studied, most of which are made from quartz (n = 78 or 92.8%), with just a few made from basalt (n = 4) or phonolite (n = 2). We have identified the use of different blanks, of which flakes were employed the most (n = 50 or 59.5%), while slabs, blocks and split blanks show a more discrete and quite similar presence (n = 12, 11, and 11, respectively) (Table A in S1 File). Blank splitting was carried out by the bipolar echnique as suggested by the presence of opposite platforms with shallow pits, opposite notches, crushing ridges and plunging and irregular fracture planes tending toward 90° [52–53]. Flakes are very often unshaped (RI 0) or slightly shaped (RI 1), whereas blocks and slabs (whole or split) tend to show a moderate (RI 2) or extensive configuration (RI3). Thereby, the unshaped or slightly shaped artifacts are the most common (RI0, n = 27 or 32.1%, and RI1 n = 32 or 38% respectively), while those with moderate or extensive configuration are represented less (RI2, n = 17 or 20.2%, and RI3 n = 8 or 9.5% respectively).

The production of large flakes is thus one of the most important technological processes employed in tool manufacturing. Flake detachment is either aligned with the longitudinal axis (n = 21) or transversal axis (n = 19) of the piece, but few pieces display an oblique flaking axis (n = 5). The butts identified in the former group are cortical (n = 9) or plain (n = 9), and the butts in the second are preferentially plain (n = 9) over cortical (n = 4). The most frequent of Toth’s flake types are Type 2 and Type 5 (n = 16 each type), Type 6 is poorly represented (n = 6) and Type 3 and Type 4 are rare (n = 2 and n = 1 respectively). The scars on the dorsal surface show a parallel (n = 17) or orthogonal (n = 10) arrangement. These large flakes are not the result of LCT shaping since only 3 LCTs display large-size scars (≥10 cm). Therefore, they probably come from the numerous large and/or giant cores documented in FLK W [3]. Indeed, a refitting between a giant core and a large flake was documented in the course of the present research.

The reduction of blanks by either retouching or shaping is the other remarkable technological process documented. Retouching is basically associated with slightly modified artifacts (RI1) and was usually conducted through unifacial isolated removals or short series of parallel removals rather than continuous (scraper-like) or bifacial removals. In contrast, shaping is registered in artifacts displaying moderate or extensive configuration (RI2 and RI3) and was generally led by series of continuous removals that modified the angle and delineation of the natural edges through secant removals that configured more obtuse working edges with indented profiles (Table B in S1 File). Although the lateral regions provide the largest dihedrals (unifacially or bifacially shaped), the most intensive volume transformation is centered on distal and proximal areas, specifically on those that show the most intensive reduction (RI3).

**Fig 5:**
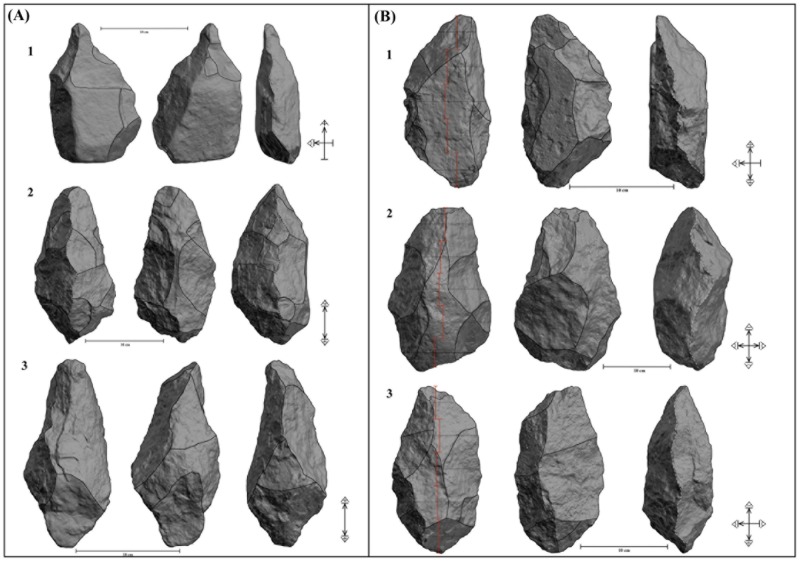
LCTs from FLK W. (A) 3D Models of 3 trihedrals and their tool functioning schemes. (B) 3D models of 3 handaxes and their tool functioning schemes (note their lack of frontal symmetry).

**Fig 6:**
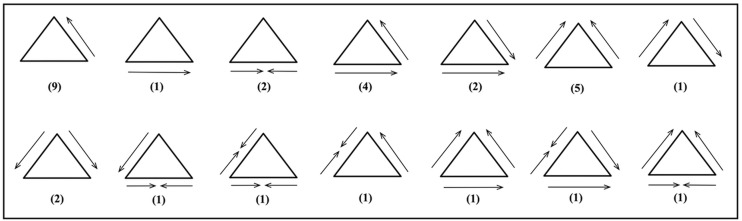
Trihedral configuration schemes.

**Fig 7:**
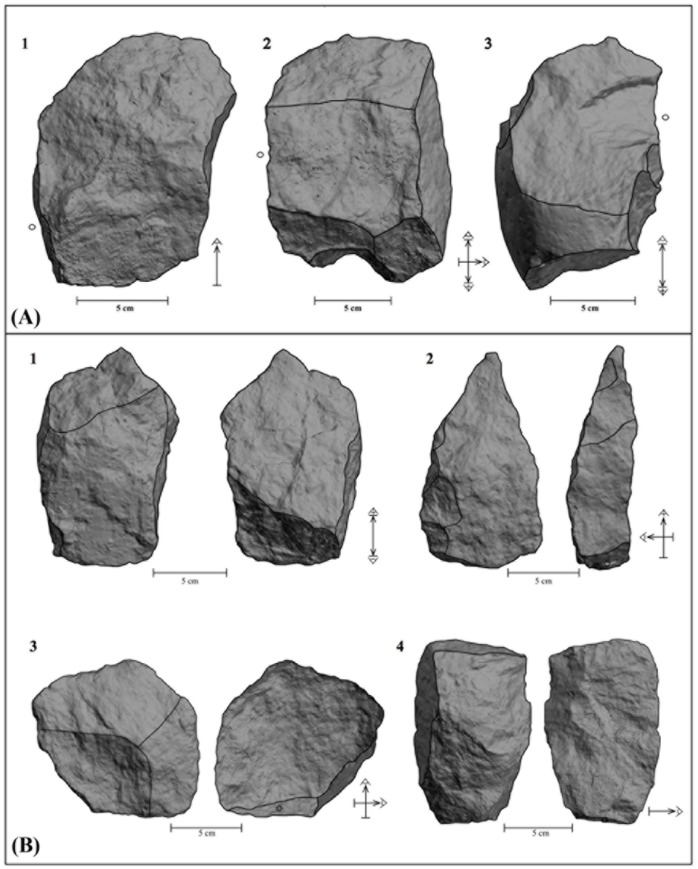
LCTs from FLK W. (A) 3D models of 3 cleavers and their tool functioning schemes (butt is indicated by a dot). (B) 3D models of RI 1 artifacts (1 and 2) and RI 0 artifacts/flakes (3 and 4) and their tool functioning schemes (RI = Reduction intensity) (butt is indicated by a dot).

**Fig 8:**
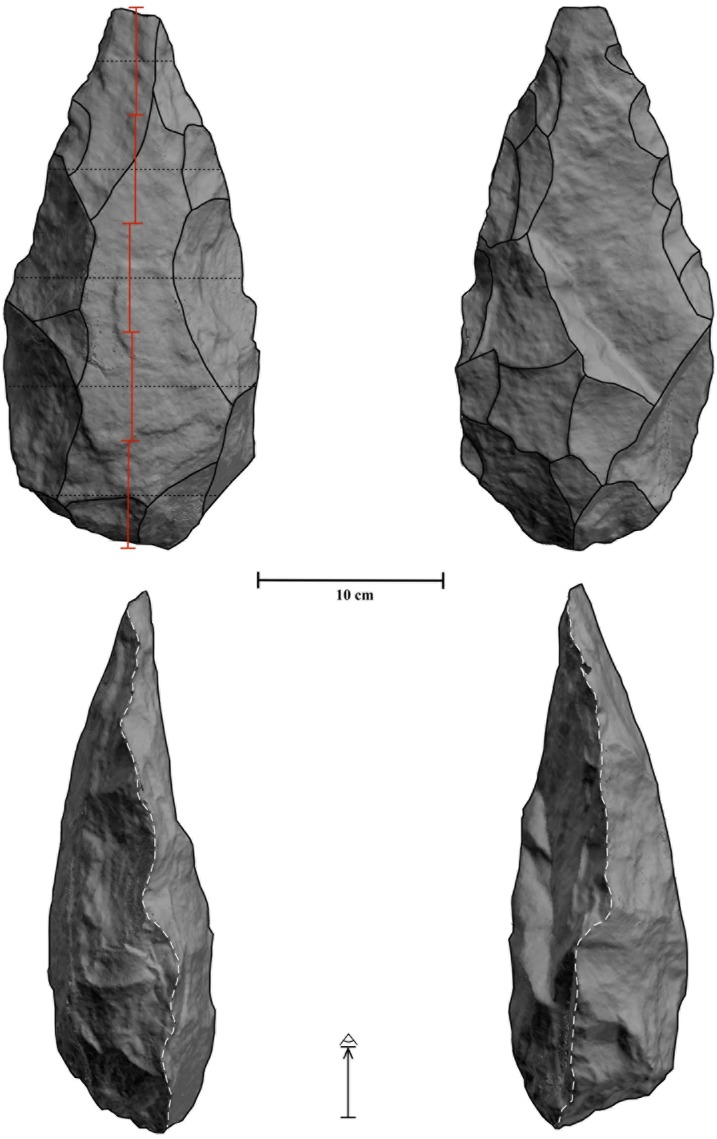
Biface from FLK W. 3D model of the refined handaxe and its tool functioning schemes. Note its accurate frontal symmetry.

Tool-types show a heteronomous representation: half of the artifacts show dihedral/s (Tp3 artifacts, n = 42), some show trihedral/s and dihedral/s (Tp2 artifacts, n = 31 or 36.9%) and the artifacts that exclusively exhibit trihedral/s are the least represented (Tp1 artifacts, n = 11 or 13%). The dihedrals and trihedrals of the shaped artifacts were knapped very heterogeneously in technical and volumetric terms. This has generated a huge diversity of shapes, very difficult to classify in formal types. Nonetheless, it is possible to recognize a crude version of the classical Acheulean morphotypes: picks, cleavers and handaxes. Pick is the most abundant (n = 13) and best performed morphotype (Fig 5A). They consist of massive tools made from quartz blocks or slabs with converging, often asymmetric sides with thick pointed tips formed by semiabrupt knapping (75–90°). Almost half of the blanks were split using bipolar technique, generating elongated pre-forms in which distal and/or proximal regions were configured trihedrals. All picks show triangular distal sections and their mesial and distal sections are more varied, but a quadrangular or rectangular shape is quite abundant in mesial regions. The picks often show a moderate reduction intensity (RII mean = 0.35), but one exhaustively manufactured pick exhibits the highest RII value (n = 1) in the studied sample (Fig 5A). Trihedrals are located on the distal and proximal regions and their configuration often required significant transformation of these segments because many of them show two or three knapped planes (Fig 6). Compared to the other morphotypes, the picks show the highest typometrical mean values and a low EI mean value (0.54) which evidences their tendency to elongation (Table C in S1 File). There are 4 flakes, two of them made from volcanic rocks that could be classified as crude cleavers since they exhibit a non-retouched transverse cutting edge at right angle to the length of the piece and formed by the intersection of the ventral face and a scar of the dorsal face (Fig 7A). In addition to the transversal dihedral, 2 cleavers display a cursorily retouched lateral dihedral and in 2 cases a distal trihedral was shaped. Compared to the other morphotypes, the 4 cleavers present the lowest typometrical, RII, OI and EI mean values (Table C in S1 File). Finally, we have identified 4 handaxes, characterized by two side dihedrals bifacially shaped that converge distally and proximally and generate robust dihedrals or trihedrals (Fig 5B). They are elongated and heavy artifacts extensively reduced (the EI mean value is 0.54) which evidence their tendency to elongation (Table C in S1 File). Planform symmetry (i.e. three-dimensional symmetry) is absent in the three handaxes made from quartz blocks or slabs and a very rude frontal symmetry can be recognized (Fig 5B). However, there is a refined handaxe made from basalt which displays both plan view symmetry and elaborate bifacial shaping and trimming (Fig 8).

#### Statistical analysis

We undertook two batteries of statistical tests to investigate the influence of specific variables on tool design (see S1 and S2 Tables). The first battery investigated the relationship among a group of variables chosen to analyze the volumetric structure of the 84 tools studied here (i.e. blank, RII, SDI, OI, EI, length, width, thickness, weight, tool-type, number of scars, and number of functional segments). We first assessed if there were significant differences in RII between the types of blanks. The assumption of normality was not met in RII (p = < 0.001, Shapiro-Wilk test) and, accordingly, we used a Kruskal-Wallis rank sum test. The results were significant (p = 0.001), indicating that the mean rank of RII was significantly different between the types of blanks (Fig A in S1 File). Since the overall test was significant, were examined pairwise comparisons between each level of blank. The results of the multiple comparisons indicated significant differences between the following pairs: flake-block, flake-slab, and flake-split (Table D in S1 File). In other words, blocks and slabs (whole or split) show the most intensive reduction, whereas flakes very often exhibit null or scarce reduction. Furthermore, the choice of blank greatly influences the final typometry and weight of tool, since as we comment below there is a strong positive correlation between the RII and the length, thickness and weight of artifacts (Fig B in S1 File).

We conducted a Pearson correlation test to observe correlation between pairs of shape and size variables (Fig B in S1 File). We used Cohen's standard to evaluate the strength of the relationships, where correlation coefficients (r) between 0.10 and 0.29 represent a small effect size, coefficients between 0.30 and 0.49 represent a moderate effect size, and coefficients above 0.50 indicate a large effect size. According to the results (Table E in S1 File), there was a significant positive correlation and large effect size between the following pairs of variables: RII and length, RII and thickness, RII and weight, RII and number of scars, RII and SDI, SDI and number of scars, length and thickness, length and weight, length and number of scars, thickness and weight, thickness and number of scars, and weight and number of scars. The following pairs of variables showed a significant positive correlation and moderate effect size: RII and OI, SDI and OI, SDI and length, SDI and thickness, SDI and weight, and OI and number of scars. There was a significant negative correlation and moderate effect size between EI and length. In sum, RII is the variable with greater influence over the rest of variables studied in this test (Table E in S1 File).

On the other hand, according to the MANOVA test conducted, the following variables RII, OI, EI, length, width, thickness, weight, number of scars, and number of functional segments showed significant differences among the levels of tool-type (i.e. Tp1-3) (p = < 0.001). We further investigated the effects of tool-type on these variables, and given that none of them show a normal distribution we used Kruskal-Wallis rank sum test (p = <0.05 in all cases, Shapiro-Wilk test). According to the results, there were significant differences in RII (p = 0.018), SDI (p = 0.017), EI (p = 0.019), length (p = 0.003), thickness (p = 0.004), weight (p = 0.014), number of scars (p = 0.018) and number of functional segments (p = < 0.001) by tool-type levels. Such differences are between Tp1 artefacts with regard to Tp2 and Tp3 artefacts (Figs C-J in S1 File).

The second statistical battery investigated the potential influence of different variables (i.e. length-edge, angle-edge, edge delineation, shaping-type, RII, tool-type) on the form of the dihedrals documented (n = 98) in the 84 tools studied. With this purpose, we first conducted a Pearson correlation test among RII, length-edge, and angle-edge (Fig K in S1 File). To evaluate the strength of the relationships we used Cohen's standard. According to the results (Table F in S1 File), there was a significant positive correlation with a moderate effect size between RII and length-edge; a significant positive correlation with a large effect size between RII and angle-edge; and a significant positive correlation with a moderate effect size between length-edge and angle-edge. These results indicated that as RII increases, length and angle-edge trend to increase.

To assess if there were significant differences in RII between the levels of shaping (i.e. unshaped, unifacial or bifacial), we employed Kruskal-Wallis rank sum test, since RII did not show a normal distribution (p = < 0.001, Shapiro-Wilk test). The results indicated that the mean rank of RII was significantly different between the levels of shaping (p = < 0.001) (Fig L in S1 File). We additionally examined pairwise comparisons between each level of shaping. The results of the multiple comparisons indicated significant differences between the following variable pairs: bifacial-unshaped and unifacial-unshaped (Table G in S1 File). These results indicated that as RII increases, the degree of edge transformation trends to increase − from unshaped to bifacial shaping.

We also conducted statistical tests to assess if there were significant differences in length-edge and angle-edge between the levels of shaping. The assumption of normality was not met in the case of length-edge (p = < 0.001, Shapiro-Wilk test), and for this reason we used a Kruskal-Wallis rank sum test. The results were not significant (p = 0.117), indicating that the mean rank of length-edge did not vary across the levels of shaping. Therefore, shaping was not employed for edge enlargement. Conversely, we employed one-way ANOVA test in the analysis of the angle-edge, since it met the assumption of normality (p = 0.446, Shapiro-Wilk test) and homogeneity of variance (p = 0.148, Levene´s test). The results of the ANOVA test indicated (p = < 0.001) that there were significant differences in angle-edge among the levels of shaping. To further examine the differences among the variables, T-tests were calculated between each pair of measurements. Tukey pairwise comparisons were conducted for all significant effects and the significant effects found were: the mean of angle-edge for bifacial shaping was significantly larger than for unshaped; the mean of angle-edge for unifacial was significantly larger than for unshaped. In other words, edge transformation trends to create more obtuse edges.

Finally, we carried out a Chi-Square test of independence to examine whether shaping-type and edge delineation are independent. The result was significant (p = < 0.001), suggesting that both variables were related to one another. The following level combinations have observed values that were greater than their expected values: unshaped-strait, unshaped-irregular, unshaped-pointed, unifacial-pointed, bifacial-convex, unshaped-concave, and unifacial-concave. The following level combinations have observed values that were less than their expected values: unifacial-strait, bifacial-strait, unifacial-irregular, bifacial-irregular, bifacial-pointed, unshaped-convex, unifacial-convex, and bifacial-concave (Table H in S1 File). These results suggest that edge transformation was probably aimed to generate different delineations from those showed in the untransformed dihedrals.

### Tool functioning

**Fig 9:**
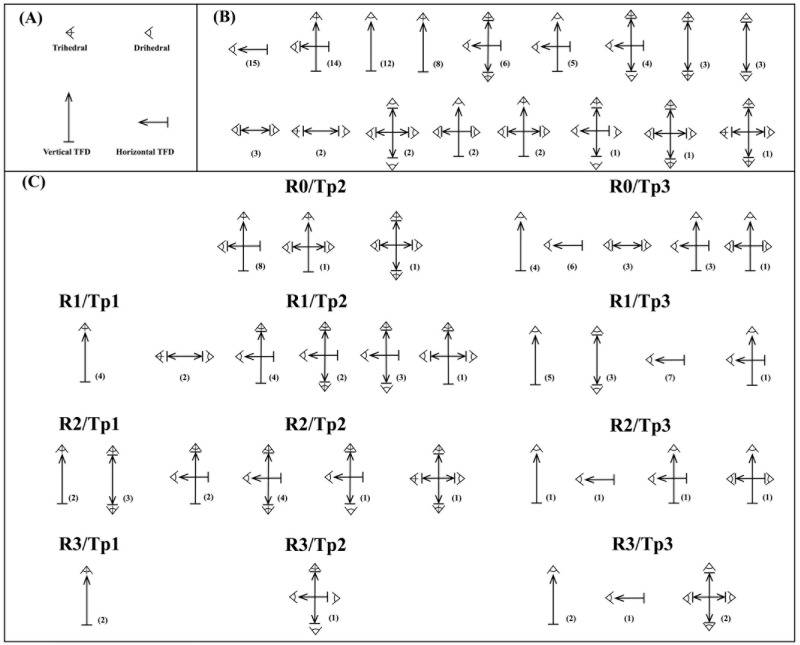
Tool functioning schemes. (A) Legend (Tool functioning direction = TFD). (B) Tool functioning schemes according to their numerical representation. (C) Tool functioning schemes sorted by reduction intensity (RI) and type of artifacts (Tp).

The 84 artifacts included in this study have been sorted into 17 tool functioning schemes (Fig 9B). The most represented schemes are: lateral dihedral (n = 15 or 17.8%); distal trihedral combined with lateral dihedral (n = 14); distal dihedral (n = 12); and distal trihedral (n = 8). Combined TFD is the most represented (n = 33 or 39%), followed by vertical TFD (n = 30 or 36%) and horizontal TFD (n = 21 or 25%). Likewise, the combined TDF displays the widest range of tool functioning schemes and is principally registered in Tp2 artifacts and some Tp3 artifacts; whereas the other types of TFD show a lesser variety of tool functioning schemes. The vertical TDF is documented in Tp1 and Tp3 artifacts, and the horizontal TFD is recorded in some Tp3 and a few Tp2 artifacts (Table I in S1 File). More specifically, the Tp1 artifacts show 2 tool functioning schemes with vertical TFD, Tp2 artifacts show 8 tool functioning schemes with either horizontal and combined TFD, and Tp3 artifacts show 7 tool functioning schemes with the 3 types of TFD (Fig 9C).

Regarding the functioning patterns of the most iconic Acheulean morphotypes: picks show two distinctive patterns, vertical TFD (single n = 6; double n = 4) and combined TFD (n = 3) (Fig 5A); the same patterns are recorded in cleavers, two were used with a vertical TFD (single n = 1; double n = 2) and the other two with a combined TFD; the three handaxes made from quartz were used with a combined TFD (Fig 5B) while the most sophisticated handaxe was employed with a vertical TFD because despite its whole being perimeter flaked, the working edge (<90°) is only located in the distal region (Fig 8).

#### Statistical analysis

**Fig 10:**
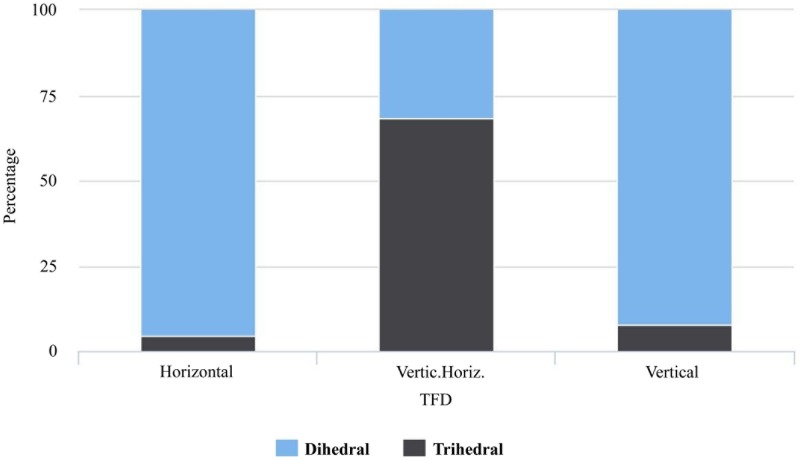
Tool functioning direction (TFD) and dihedral geometry. Bar plot of TFD grouped by geometry.

We undertook two sets of statistical analysis to assess the functional capabilities of the studied tools (tool functioning), enabling us to suggest hypothetical patterns of use and action according to the loading force they could apply (see S3 and S4 Tables). The first analytical set investigated the relationship in the 84 artifacts studied between the variables related with tool functioning (i.e. TFD and action) and a group of variables related with volumetric structure (i.e. blank, geometry, tool-type, RII, OI, EI, and weight). We first focused on the relationship between TFD (i.e. horizontal, vertical, and combined) and the qualitative variables of the second group: blank (i.e. flake, block, slab, and split), geometry (i.e. dihedral and trihedral) and tool-type (i.e. Tp1-3), and with this proposal we applied the Chi-Square Test of independence. The results indicated that TFD and tool-type were related to one another (p = < 0.001), as well as TFD and geometry (p = < 0.001). However, TFD and blank could be independent of one another (p = 0.162). The following combinations between TFD and tool-type were greater than their expected values: vertical-Tp1, combined-Tp2, and horizontal-Tp3. Conversely, the following combinations showed lesser values than their expected values: horizontal-Tp1, combined-Tp1, vertical-Tp2, horizontal-Tp2, vertical-Tp3, and combined-Tp3 (Table J in S1 File). According to the significant relationship between TFD and geometry, the following combinations were greater than their expected values: vertical-dihedral, horizontal-dihedral, and combined-trihedral (Fig 10). In contrast, the following combinations showed lesser values than their expected values: combined-dihedral, vertical-trihedral, and horizontal-trihedral (Table K in S1 File).

On the other hand, we conducted a MANOVA test to assess if there were significant differences in the linear combination of RII, OI, and EI between the levels of TFD. The result was significant (p = < 0.001), suggesting a linear combination of RII, OI, and EI among the levels of TFD. To further investigated the effects of TFD on these quantitative variables, we performed Kruskal-Wallis rank sum tests since none of them met the assumption of normality (p = < 0.05 in all cases, Shapiro-Wilk test). According to the results, there were significant differences in RII (p = 0.019), OI (p = < 0.001), and EI (p = 0.020) by the levels of TFD (Figs M-O in S1 File). Pairwise comparisons were examined RII, OI, and EI among the levels of TFD. The following pairs showed significant differences: horizontal-combined for the mean rank of RII, vertical-combined for the mean rank of OI, and horizontal-combined for the mean rank of EI (Tables L-N in S1 File).

**Fig 11:**
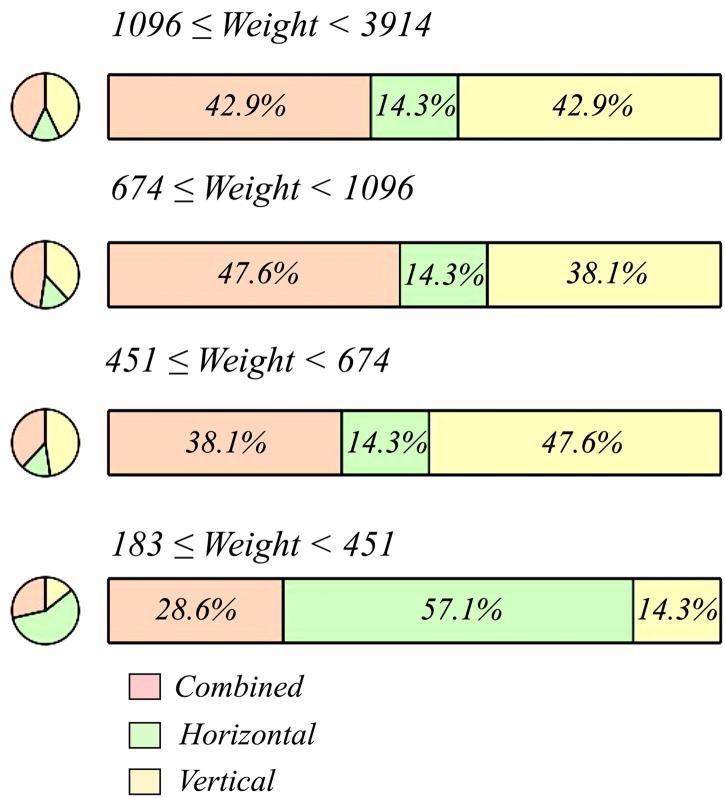
Tool functioning direction (TFD) and weight. Bar plot of TFD grouped by quartiles of weight (in grams).

In the same vein, we assessed if there were significant differences in weight between the levels of TFD. The assumption of normality was not met in RII (p = < 0.001, Shapiro-Wilk test) and, consequently, we used the Kruskal-Wallis rank sum test. The results were significant (p = 0.004) and thus the mean rank of weight was significantly different between the levels of TFD (Fig P in S1 File). The pairwise comparisons examined between each level of TFD indicated significant differences between the following variable pairs: vertical-horizontal and horizontal-combined (S Table O in S1 File). In other words, the distribution of weight varies across values of TFD (p = 0.009, Kolmogorov-Smirnov test). For instance: 57.1% of the artifacts employed horizontally are concentrated in the first quartile (<451 g), whereas just 14.3% of the artifacts employed vertically are in the first quartile (Fig 11). This disparity is very suggestive, as tool mass is directly connected to the loading force and is therefore a crucial variable to infer tool functional capabilities (i.e. action).

In response to this observation, the 84 artifacts have been divided into two weight groups (group 1 <500g; group 2 ≥500g). The results of the Chi-square test suggest that TFD and weight group are related to one another (p = < 0.001). The following combinations between TFD and weight group were greater than their expected values: vertical-group 1, horizontal-group 1, and combined-group 2. Conversely, the following combinations showed lesser values than their expected values: combined-group 1, vertical-group 2, and horizontal-group 2 (Table P in S1 File). More precisely, horizontal TFD accumulates 52.2% of the objects included within group 1, whereas just 13.8% of the artifacts in group 2 show horizontal TFD. In the light of these results, we can infer changes in the functional capabilities (action) of the tools below or above 500 g. To be more precise, the mass threshold should be situated in the second quartile (≥451-<674 g) according to the statistical test conducted to observe the distribution of weight across values of TFD (Fig 11). The resting/thrusting threshold can be situated around this mass value, which means that below or above the threshold (≥451-<674 g) cutting efficiency could decrease significantly and, in turn, a change of action would be required. It is quite likely that within this mass threshold both actions could be implemented efficiently. In sum, we can distinguish three groups of artifacts according to the action they might have performed: the artifacts whose mass is <451g would be used in resting actions (n = 21 or 25%), the artifacts whose mass is between ≥451-<674 g would be used in resting and/or thrusting (i.e. combined actions) (n = 21 or 25%), and the artifacts whose mass is >674 g would be used in thrusting actions (n = 52 or 50%).

**Fig 12:**
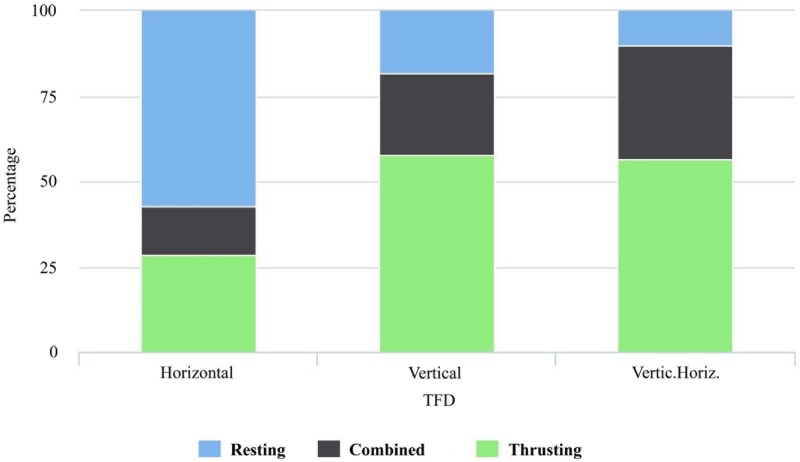
Tool functioning direction (TFD) and action. Bar plot of TFD grouped by action.

We carried out Chi-square tests to examine whether action and the following qualitative variables were independent: tool-type, TFD and blank. The results indicated that action and tool-type could be independent of one another (p = 0.198); whereas action and TFD (p = 0.003) (Fig 12), and action and blank are related to one another (p = < 0.001). The following combinations between action and TFD were greater than their expected values: thrusting-vertical, resting-horizontal, combined-combined, and thrusting-combined. The following combinations showed lesser values than their expected values: resting-vertical, combined-vertical, combined-horizontal, thrusting-horizontal, and resting-combined (Table Q in S1 File). The following combinations between action and blank were greater than their expected values: resting-flake, combined-flake, thrusting-block, thrusting-slab, and thrusting-split. The following level combinations had observed values that were less than their expected values: thrusting-flake, resting-block, combined-block, resting-slab, combined-slab, resting-split, and combined-split (Table R in S1 File).

We also examined whether there were significant differences in weight, RII, OI, and EI among the levels of action. Owing to these qualitative variables not meeting the assumption of normality (p = <0.05 in all cases, Shapiro-Wilk test), we used the Kruskal-Wallis rank sum test. According to the results, the mean rank of OI (p = 0.073) and EI (p = 0.314) was similar for each level of action. However, the mean rank of weight (p = <0.001) was significantly different between the levels of action (Fig Q in S1 File). The pairwise comparisons examined between each level of action indicated significant differences between the following variable pairs: resting-combined, resting-thrusting, and combined-thrusting (Table S in S1 File). Likewise, the mean rank of RII (p = < 0.001) was significantly different between the levels of action (Fig R in S1 File). The pairwise comparisons examined between each level of action indicated significant differences between the following variable pairs: resting-thrusting and combined-thrusting (Table T in S1 File).

The second set of statistical analysis was aimed at investigating the relationship between the variables related with tool functioning (i.e. TFD and action) and a group of variables (i.e. edge angle, length and form) related with the edge form of 98 dihedrals recorded in the 84 artifacts studied. Chi-Square tests were conducted to examine whether TFD and action were independent in relation to delineation. The results indicated that action and delineation could be independent of one another (p = 0.223), but TFD and delineation are related to one another (p = 0.002). The following combinations between TFD and delineation were greater than their expected values: vertical-straight, horizontal-irregular, vertical-pointed, and vertical-convex. Conversely, the following combinations showed lesser values than their expected values: horizontal-straight, and vertical-irregular (Table U in S1 File).

**Fig 13:**
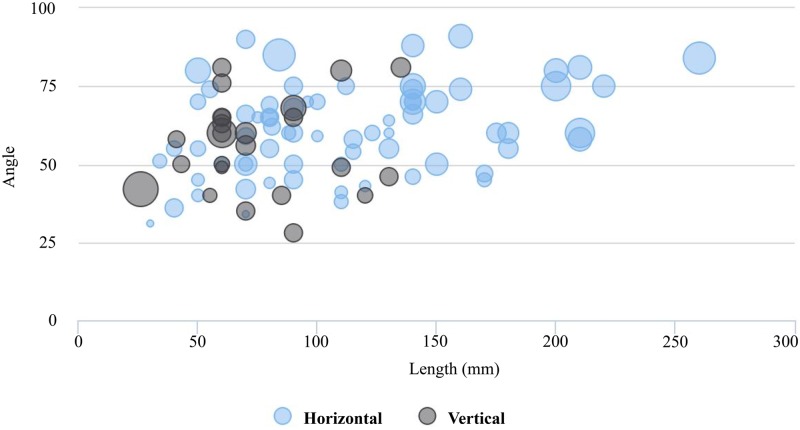
Dihedral functioning according to tool functioning direction (TFD). Bubbleplot of edge length and angle weighted by weighted and grouped by TFD (mm = millimeters).

**Fig 14:**
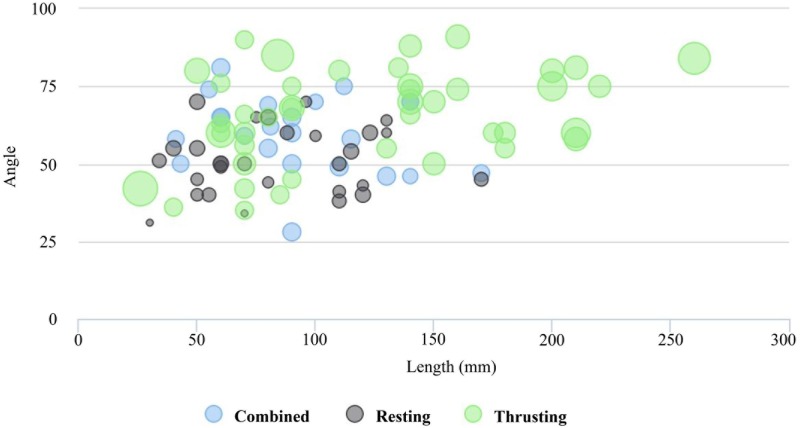
Dihedral functioning according to action. Bubbleplot of edge length (in millimeters) and angle weighted by weighted and grouped by action (mm = millimeters).

Finally, we assessed if there were significant differences in length-edge and angle-edge between the levels of TFD (i.e. horizontal and vertical) and action (i.e. resting, combined, and thrusting). The assumption of normality and homogeneity was evaluated first. The variable angle-edge met both assumptions (p = 0.499, Shapiro-Wilk test; p = 0.390, Levene´s test), but length-edge showed a non-parametric result (p = < 0.001, Shapiro-Wilk test). Thereby, one-way ANOVA test was conducted to determine whether there were significant differences in angle by action and TFD. According to the results there were significant differences in angle-edge among the levels of action (p = < 0.001) and TFD (p = 0.025). To further examine the differences among the variables, T-tests were calculated between each pair of measurements. Tukey pairwise comparisons were conducted for all significant effects. For the main effect of action, the mean of angle-edge for resting action was significantly smaller than for combined and thrusting actions; and for the main effect of TFD, the mean of angle-edge for horizontal TFD was significantly larger than for vertical TFD. On the other hand, the non-parametric test of Kruskal-Wallis was conducted to assess if there were significant differences in length between the levels of TFD. The results indicated that the mean rank of length-edge was significantly different between the levels of TFD (p = < 0.001) (Fig S in S1 File), and between the levels of action (p = 0.017) (Fig T in S1 File). Pairwise comparisons were examined between each level of action, indicating significant differences between resting-thrusting (Table W in S1 File). Summarizing, the dihedrals that were used with a horizontal functioning direction tend to show larger and more obtuse edges than those that were used with a vertical functioning direction (Fig 13), and the tools whose dihedrals have been employed in thrusting actions tend to show larger and more obtuse edges and higher loading force than those employed in resting actions (Fig 14).

### Tool production

The aim of this section is to gather, synthesize, order and relate the results described above, in order to obtain a clear vision of the tool production processes. The primary division between the two production processes identified here is based on the type of blank used. There is a strong statistical correlation between blank and RII. Likewise, the RII is correlated with many other variables, namely: number of scars, SDI, length, thickness, weight, OI, number of operative segments, working edge properties, TFD and action. Blank choice is therefore a critical decision since it determines very relevant elements of tool production. In the light of these results, it is possible to distinguish two production processes: one based on the production of large flakes, and the other on the configuration of large and heavy blocks or slabs (whole or split).

The former process is quantitatively the most important. Some flakes are cursorily retouched by unifacial removals isolated or grouped in short series of parallel removals rather than by continuous (scraper-like) or bifacial removals. Compared with the tools of the other production process, these stone tools display a lower operative scope, their dihedrals tend to be shorter, more acute (a sharp edge), with straight delineation and were likely used with a horizontal TFD and resting actions. The sharp and straight edges of the large flakes concentrates greater cutting stresses over smaller surface areas, which means that cutting is achievable with application of less force, facilitating quicker and larger fracture initiations and the cutting of materials of greater strength (i.e. resistance). The greater cutting consistency of these edges is particularly suited for performing long cuts through large portions of resistant, extensible and flexible materials (e.g. plant and animal tissue) with a relatively limited force, finesse, precision and time of use [28].

The other production process consists of large and heavy blocks or slabs (whole or split) on which working edges (dihedrals and trihedrals) were shaped. Shaping was generally led by series of continuous removals that modified the angle and delineation of the natural edges, and through secant removals that configured more obtuse working edges with varied delineations. The lateral regions provide the largest dihedrals (unifacially or bifacially shaped), but the most intensive volume transformation is centered on distal and proximal areas, specifically on those that show most intensive reduction (RI3). In contrast with the former group, these objects have more operative segments and their dihedrals are longer, more obtuse, bifacially shaped and with indented profiles. The detrimental effect of more obtuse edge angles (i.e. >50°) could be counteracted by increasing loading force through thrusting actions. The irregular (i.e. sinuous) profile of these tools would help reduce the speed of blunting and allow activities of longer duration to be undertaken [28]. This may explain why hominins would expend greater allocations of time and energy into shaping these artifacts when a flake of equal size and mass might suffice [29]. The incipient standardized manufacture that these artifacts displayed was not accompanied by the standardization of shape or plan form symmetry, with the exception of a magnificently manufactured handaxe. These large and heavy artifacts made on blocks or slabs (whole or split), which often present a moderate or extensive configuration (RI2-3 or RII = ≥ 0.25), show the following significant trends towards: tool-mass (significant statistical correlation between RII and weight: p = <0,001, r = 0,44, Pearson Correlation text), tool elongation (significant statistical correlation between RII and EI: p = 0,047, r = -0,22, Pearson Correlation text), vertical TFD (in combination with horizontal TFD or not) (see Fig M in S1 File) and thrusting action (see Fig R in S1 File). The aggregation of these four elements increases the tool loading force dramatically. It is thus obvious that the main objective of this production process was to manufacture artifacts with a huge potential loading force that would be employed in heavy-duty tasks in which greater application of force is required.

## Discussion

There are a number of ecological and biological factors that might make the emergence of the Acheulean possible. The coincidence in time (c. 1.7 Ma) of a long-term drying period, with the consequent expansion of grasslands and grazing bovid species in east Africa, and the occurrence of the earliest Acheulean would suggest that a climate change triggered this technological change [54–56]. Changes in brain structure and function in early Homo could also support this technological change since the Acheulean was more demanding cognitively than the Oldowan [57]. Likewise, changes in the upper limb of the early Homo were also required due to the greater manipulative forces that the Acheulean knappers had to exert and resist [44, 50, 58–59]. It has also been suggested that the appearance of modern human-like hands is associated with the appearance of the Acheulean [48–60]. Recent fossil discoveries in east African sites have demonstrated that modern human-like hands and post-cranial anatomy emerged shortly before, or coincident with, the earliest Acheulean [61–63].

Although these factors provided a very suggestive context to explain “why” such a technological change may occur, they cannot explain “how” a core-and-flake industry gave way to a technology based on the production of large and heavy shaped tools. Indeed, what were the economic mechanisms that triggered such remarkable changes in hominin technological behavior? In our opinion, to answer these question, we first have to clarify if LCTs “at the moment of innovation represented new tools for performing long lasting tasks (such as butchery) or whether new tasks were added to the behavioral, adaptive repertoire” (see page 50 in [63]. In other words, were the new tools linked to new tasks?

Direct evidence supporting the function of the early African Acheulean tools is very scarce and use-wear and residues analyses are not yet available. To date, the confirmed functional application of the early Acheulean LCTs is plant processing activities, particularly chopping acacia wood [64]. On the other hand, an increasing number of current investigations are providing compelling evidence of large herbivore carcass exploitation through the use of handaxes during the Middle Pleistocene [65–67]. Likewise, numerous experiments have investigated the comparative functional capabilities of flakes and handaxes as cutting tools in the context of butchery with asymmetric results: some authors highlighted the effectiveness of flakes in the whole butchery procedure [68–70], while others concluded that handaxes are best for heavy duty butchery (split-carcass) and to cut through large portions of flesh [71–74]. It is necessary to note from these experimental handaxes are different in terms of manufacture, size and raw material that those recovered in FLK W. Although this East African site has provided the earliest firm evidence of Acheulean tools occurring spatially and functionally associated with the exploitation of herbivore carcasses of various sizes [3], this neither demonstrates nor refutes that LCTs were used in butchery. In sum, a functional study (i.e. use-wear and residue analysis) would be required to test plant-processing hypothesis or carcass-butchery hypothesis.

The results achieved in this scientific paper are unable to support either of these two hypotheses. However, it can contribute a substantial empirical framework to argue the functional contexts in which the earliest Acheulean tools could be used. Accordingly, these tools were likely to have been employed in activities with different duration, to transform materials with different resistance, and in a context of an increasing demand of loading force. Therefore, the earliest Acheulean tools were developed in a diverse functional context, where the demand of loading force seems to trigger their appearance. The diverse functional context in which the new large toolkit (i.e. LCTs and large flakes) would be involved is not exclusively the breakthrough that would lead to the Oldowan-Acheulean transition. Actually, in our opinion, what would be a revolutionary innovation is that the new tools extended the operating scope of the toolkit of hominins in terms of cutting efficiency, action, load force application, activity duration, and materials to transform. This would allow ‘new tasks’ to be undertaken and increase efficiency in ‘old tasks’ (i.e. butchery). However, what is most surprising is that the handaxe was conceptually and technically defined from the very beginning of the Acheulean, as suggested by the basalt handaxe unearthed at FLK W, although mysteriously refined handaxes were probably rarely knapped during the early Acheulean (>1.3 ma) [1–12]. This seems to confirm that the decision processes underlying the production of the earliest Acheulean tools are functional in character rather than aesthetic.

## Conclusions

Climatic and biological changes that occurred shortly before, or coincident with, the Acheulean emergence might have enabled it. This technological change may thus be understood as part of a major adjustment in the economic structure that some groups of hominins, with modern human-like hands [48], employed to adapt to a new ecological scenario. According to the production processes identified here, the earliest Acheulean economy involved diversified functional situations in which tasks with variable durations were carried out to transform resources with different resistances. The exploitation of large and resistant resources suggest that the main economic mechanism that governed the emergence of the Acheulean toolkit is an increase in the demand of work load. The results of a current experimental study strengthen this conclusion [47]. The new Acheuelan tools were designed for this purpose, extending the operating scope of the hominin’s toolkit in terms of cutting efficiency, force application, activity duration, materials to transform and tasks to perform. The technological change would be able to satisfy this demand in order to carry out new tasks and/or increase the efficiency of old tasks. In sum, the most important outcomes of the Acheulean emergence seems to be: diet diversification, novel feeding patterns, more effective acquisition and/or consumption of food resources, and novel food processing technology and extractive tools.

The decision processes underlying the production of the earliest Acheulean tools have an evident functional character. The elements of design form in handaxes (i.e. tool elongation, support for working edge, lateral extension and thickness adjustment, according to [75]) are present in the best-shaped tools at FLK W, but nevertheless are poorly standardized. According to Chevrier (see page 730 in [4], this may respond to a phenomenon of invention in which design (volumetric construction) was subordinated to functional parameters rather than structured plan forms. Astonishingly, in this primitive stage, the iconic handaxe design form was conceptually and technological defined. If we accept that KS4 and KGA6-A1 are “significantly” older than FLK W, this iconic design would have been achieved in <100 Ky. Be that as it may, in the light of the current evidence, its development was not as gradual as previously thought [2, 5, 21]. In our opinion, the question is not whether there was an evolution in the Acheulean towards more refined and symmetrical handaxes, the point is why sophisticated handaxes are so rare during the early Acheulean and then become more common [19–20].

We would like to conclude this paper by highlighting the huge potential that mechanical and ergonomic investigation has in our understanding of hominin techno-economic behavior [28]. Specifically, it is of great interest for the Oldowan-Acheulean gradient, since it has the potential to explain why tool sizes and shapes varied within and between assemblages. On the basis of an original techno-functional approach we have provided a complete description of the origin of the Acheulean tools in order to reach the economic mechanisms that may trigger such remarkable changes in hominin technological behavior. Future experiments are needed to investigate tool functioning with large and heavy cutting tools further, especially to explore the relationships between edge form, mass, action and TFD. All in all, we have tried to place the focus of the Oldowan-Acheulean gradient on the emerging economy rather than on the emergence of iconic morphotypes.

## Supporting information

S1 File Results of the statistical tests. **Table A. Blanks**. Types of blanks sorted by types of reduction intensity (RI) and type of artifact (Tp). (b/s = block/slab).
**Table B. Dihedrals**. Edges attributes sorted by types Reduction Intensity (RI: RI0 = 0.00; RI1 = 0.25; RI2 = ≥0.25 <0.50; RI3 = ≥0.50–1) and Operative Index (OI) (mn = mean; u = unmodified; m = modified; dentic. = denticulated).
**Table C. Morphotypes**. Number of objects, scars, indexes (RII = Reduction Intensity Index; OI = Operative Index; EI = Elongation Index) shorted by morphotypes (Thickn. = Thickness).
**Table D. Post-hoc test between RII and blank**. Pairwise comparisons for the mean ranks of RII by levels of blank.
**Table E. Pearson Correlation Matrix among RII, OI, EI, length, width, thickness, weight, and number of scars**. Statistically significant correlation were observed in accordance with Bonferroni correction and were only determined if p = ≤ 0,006 (p = p-value; r = correlation coefficient; NC = no correlation or p-value > 0.006).
**Table F. Pearson Correlation Matrix among RII, length-edge, and angle-edge**. Statistically significant correlation were observed in accordance with Bonferroni corrections and were only determined if p = ≤ 0,025 (p = p-value; r = correlation coefficient).
**Table G. Post-hoc test between RII and shaping**. Pairwise comparisons for the mean ranks of RII by levels of shaping.
**Table H. Chi-square test between shaping and delineation**. Observed and expected frequencies by shaping and delineation. Items in brackets represent expected cell frequencies.
**Table I. Tool functioning direction (TFD)**. Number of objects, tool functioning schemes, weight and types of reduction intensity (RI) sorted by type of TFD (M.) and artifact type (Tp) (Minim. = minimum; maxim. = maximum; Std. D. = standard deviation).
**Table J. Chi-square test between tool functioning direction (TFD) and tool-type**. Observed and expected frequencies by TFD and tool-type. Items in brackets represent expected cell frequencies.
**Table K. Chi-square test between tool functioning direction (TFD) and edge geometry**. Observed and expected frequencies by TFD and geometry. Items in brackets represent expected cell frequencies.
**Table L. Post-hoc test between RII and tool functioning direction (TFD)**. Pairwise comparisons for the mean ranks of RII by levels of TFD.
**Table M. Post-hoc test between OI and tool functioning direction (TFD)**. Pairwise comparisons for the mean ranks of OI by levels of TFD.
**Table N. Post-hoc test between EI and tool functioning direction (TFD)**. Pairwise comparisons for the mean ranks of EI by levels of TFD.
**Table O. Post-hoc test between weight and tool functioning direction (TFD)**. Pairwise comparisons for the mean ranks of weight by levels of TFD.
**Table P. Chi-square test between tool functioning direction (TFD) and weight group**. Observed and expected frequencies by TFD and weight group.
**Table Q. Chi-square test between action and tool functioning direction (TFD)**. Observed and expected frequencies by action and TFD. Items in brackets represent expected cell frequencies.
**Table R. Chi-square test between action and blank**. Observed and expected frequencies by action and blank. Items in brackets represent expected cell frequencies.
**Table S. Post-hoc test between weight and action**. Pairwise comparisons for the mean of weight by levels of action.
**Table T. Post-hoc test between RII and action**. Pairwise comparisons for the mean ranks of RII by levels of action.
**Table U. Chi-square test between tool functioning direction (TFD) and delineation**. Observed and expected frequencies by TFD and delineation. Items in brackets represent expected cell frequencies.
**Table W. Post-hoc test between length-edge and action**. Pairwise comparisons for the mean ranks of length-edge by levels of action.
**Fig A**. **Mean rank of RII by blank**. Boxplots of the ranked values of RII by the levels of blank.
**Fig B. Correlation between pairs of variables of tool shape and size**. Scatterplot matrix among RII, SDI, OI, EI, length, width, thickness, weight, and number of scars.
**Fig C. Mean rank of RII by tool-type**. Boxplots of the ranked values of RII by the levels of tool-type.
**Fig D. Mean rank of SDI by tool-type**. Boxplots of the ranked values of SDI by the levels of tool-type.
**Fig E. Mean rank of OI by tool-type**. Boxplots of the ranked values of OI by the levels of tool-type.
**Fig F. Mean rank of EI by tool-type**. Boxplots of the ranked values of EI by the levels of tool-type.
**Fig G. Mean rank of length by tool-type**. Boxplots of the ranked values of length by the levels of tool-type.
**Fig H. Mean rank of thickness by tool-type**. Boxplots of the ranked values of thickness by the levels of tool-type.
**Fig I. Mean rank of weight by tool-type**. Boxplots of the ranked values of weight by the levels of tool-type.
**Fig J. Mean rank ofnumber of scars by tool-type**. Boxplots of the ranked values of number of scars by the levels of tool-type.
**Fig K**. **Correlation between pairs of variables of RII and cutting-edge variables**. Scatterplot matrix among RII, length-edge, and angle-edge.
**Fig L. Mean rank of RII by shaping type**. Boxplots of the ranked values of RII by the levels of shaping.
**Fig M. Mean rank of RII by tool functioning direction (TFD)**. Boxplots of the ranked values of RII by the levels of TFD.
**Fig N. Mean rank of OI by tool functioning direction (TFD)**. Boxplots of the ranked values of OI by the levels of TFD.
**Fig O. Mean rank of EI by tool functioning direction (TFD)**. Boxplots of the ranked values of EI by the levels of TFD.
**Fig P. Mean rank of weight by tool functioning direction (TFD)**. Boxplots of the ranked values of weight by the levels of TFD.
**Fig Q. Mean rank of weight by action**. Boxplots of the ranked values of weight by the levels of action.
**Fig R. Mean rank of RII by action**. Boxplots of the ranked values of RII by the levels of action.
**Fig S. Mean rank of length-edge by tool functioning direction (TFD)**. Boxplots of the ranked values of length by the levels of TFD.
**Fig T. Mean rank of length-edge by action**. Boxplots of the ranked values of length by the levels of action.
(DOCX)

S1 Table Tool design: Shape and size. (XLS)

S2 Table Tool design: Dihedrals. (XLS)

S3 Table Tool functioning: Shape and size. (XLS)

S4 Table Tool functioning: Dihedrals. (XLS)
