# Supplementary material for: The origin of the Acheulean. Techno-functional study of the FLK W lithic record (Olduvai, Tanzania)
Source: PLoS One. 2017 Aug 2;12(8):e0179212. doi: 10.1371/journal.pone.0179212 (PMC5540295; doi:10.1371/journal.pone.0179212)
Supplement: S1 File — Table A. Blanks. Types of blanks sorted by types of reduction intensity (RI) and type of artifact (Tp). (b/s = block/slab). Table B. Dihedrals. Edges attributes sorted by types Reduction Intensity (RI: RI0 = 0.00; RI1 = 0.25; RI2 = ≥0.25 <0.50; RI3 = ≥0.50–1) and Operative Index (OI) (mn = mean; u = unmodified; m = modified; dentic. = denticulated). Table C. Morphotypes. Number of objects, scars, indexes (RII = Reduction Intensity Index; OI = Operative Index; EI = Elongation Index) shorted by morphotypes (Thickn. = Thickness). Table D. Post-hoc test between RII and blank. Pairwise comparisons for the mean ranks of RII by levels of blank. Table E. Pearson Correlation Matrix among RII, OI, EI, length, width, thickness, weight, and number of scars. Statistically significant correlation were observed in accordance with Bonferroni correction and were only determined if p = ≤ 0,006 (p = p-value; r = correlation coefficient; NC = no correlation or p-value > 0.006). Table F. Pearson Correlation Matrix among RII, length-edge, and angle-edge. Statistically significant correlation were observed in accordance with Bonferroni corrections and were only determined if p = ≤ 0,025 (p = p-value; r = correlation coefficient). Table G. Post-hoc test between RII and shaping. Pairwise comparisons for the mean ranks of RII by levels of shaping. Table H. Chi-square test between shaping and delineation. Observed and expected frequencies by shaping and delineation. Items in brackets represent expected cell frequencies. Table I. Tool functioning direction (TFD). Number of objects, tool functioning schemes, weight and types of reduction intensity (RI) sorted by type of TFD (M.) and artifact type (Tp) (Minim. = minimum; maxim. = maximum; Std. D. = standard deviation). Table J. Chi-square test between tool functioning direction (TFD) and tool-type. Observed and expected frequencies by TFD and tool-type. Items in brackets represent expected cell frequencies. Table K. Chi-square test between tool [file pone.0179212.s001.docx]

**S1 Results of the statistical tests**

|  | **Flake** | **Slab** | **Block** | **Spitted b/s** |  |  | **Flake** | **Slab** | **Block** | **Spitted b/s** |
| --- | --- | --- | --- | --- | --- | --- | --- | --- | --- | --- |
| **RI 0** | 17 | 0 | 0 | 0 |  | **RI 2** | 2 | 7 | 2 | 6 |
| **Tp1** | - | - | - | - |  | **Tp1** | - | - | 1 | 4 |
| **Tp2** | 10 | - | - | - |  | **Tp2** | 1 | 4 | 1 | 2 |
| **Tp3** | 17 | - | - | - |  | **Tp3** | 1 | 3 | - | - |
| **RI 1** | 20 | 5 | 4 | 3 |  | **RI 3** | 1 | 0 | 5 | 2 |
| **Tp1** | 1 | 1 | 1 | 1 |  | **Tp1** | - | - | 2 | - |
| **Tp2** | 10 | 2 | - | - |  | **Tp2** | - | - | 1 | - |
| **Tp3** | 9 | 2 | 3 | 2 |  | **Tp3** | 1 | - | 2 | 2 |

**Table A. Blanks**. Types of blanks sorted by types of reduction intensity (RI) and type of artifact (Tp). (b/s = block/slab).

|  | **Edge (u / m)** | | **Faciality (u / m)** | | **Delineation (u / m)** | | | | |
| --- | --- | --- | --- | --- | --- | --- | --- | --- | --- |
|  | **Length (mn)** | **Angle (mn)** | **Unifacial** | **Bifacial** | **Straight** | **Irregular** | **Dentic.** | **Pointed** | **Convex** |
| **RI 0** | | | | | | | | | |
| **Tp 2** | 102,08 / − | 50,92 / − | 12 / − | − /− | 10 / − | 1 / − | − /− | − /− | 1 / − |
| **Tp3** | 86,96 / − | 51,08 / − | 25 / − | − /− | 24 / − | − /− | − /− | − /− | 1 / − |
| **RI 1** | | | | | | | | | |
| **Tp 2** | 63 / 102,55 | 38,60 / 57,36 | 5 / 9 | − / 4 | 5 / 6 | − / 2 | − / 1 | − / 1 | − / 1 |
| **Tp3** | 60 / 92,09 | 60 / 60,83 | 3 /13 | − / 3 | 3 / 4 | − / 4 | − / 3 | − / 5 | − / 2 |
| **RI 2** | | | | | | | | | |
| **Tp 2** | − / 119,55 | − / 74 | − / 4 | − / 5 |  | − / 7 | − /− | − / 1 | − / 2 |
| **Tp3** | − / 122,83 | − / 61,50 | − / 5 | − / 1 | − / 2 | − / 2 | − /− | − /− | − / 1 |
| **RI 3** | | | | | | | | | |
| **Tp 2** | − / 180 | − / 72,5 | − /− | − / 2 | − /− | − /− | − /− | − /− | − / 2 |
| **Tp3** | − / 126,26 | − / 71,38 | − /− | − / 8 | − / 1 | − / 2 | − /− | − /− | − / 6 |

**Table B. Dihedrals**. Edges attributes sorted by types Reduction Intensity (RI: RI0 = 0.00; RI1 = 0.25; RI2 = ≥ 0.25 < 0.50; RI3 = ≥ 0.50-1) and Operative Index (OI) (mn = mean; u = unmodified; m = modified; dentic. = denticulated).

|  | **Nº** | **Nº Scars**  **(mean)** | **Indexes (mean)** | | | **Typometry (mean)** | | | |
| --- | --- | --- | --- | --- | --- | --- | --- | --- | --- |
|  |  |  | **RII** | **OI** | **EI** | **Length** | **Width** | **Thickness** | **Weight** |
| **Pick** | 13 | 11,2 | 0,35 | 0,42 | 0,54 | 176,6 | 92,4 | 69,4 | 1413,3 |
| **Handaxe** | 4 | 26 | 0,71 | 0,71 | 0,52 | 220,5 | 114,2 | 73,7 | 2216,5 |
| **Cleaver** | 4 | 1,75 | 0,1 | 0,31 | 0,7 | 125 | 88,2 | 44,2 | 643 |

**Table C. Morphotypes**. Number of objects, scars, indexes (RII=Reduction Intensity Index; OI=Operative Index; EI=Elongation Index) shorted by morphotypes.

| **Comparison** | **Observed Difference** | **Critical Difference** |
| --- | --- | --- |
| **Flake-Block** | 34.46 | 21.47 |
| **Flake-Slab** | 37.96 | 21.47 |
| **Flake-Splitted** | 35.11 | 20.08 |
| **Block-Slab** | 3.50 | 27.44 |
| **Block-Splitted** | 0.65 | 26.36 |
| **Slab-Splitted** | 2.85 | 26.36 |

**Table D.** **Post-hoc test between RII and blank**. Pairwise comparisons for the mean ranks of RII by levels of blank.

| **Variable** | **1** | | **2** | | **3** | | **4** | | **5** | | **6** | | **7** | | **8** | | **9** | |
| --- | --- | --- | --- | --- | --- | --- | --- | --- | --- | --- | --- | --- | --- | --- | --- | --- | --- | --- |
|  | **p** | **r** | **p** | **r** | **p** | **r** | **p** | **r** | **p** | **r** | **p** | **r** | **p** | **r** | **p** | **r** | **p** | **r** |
| **1. RII** | - | - | - | - | - | - | - | - | - | - | - | - | - | - | - | - | - | - |
| **2. SDI** | 0.001 | 0.68 | - | - | - | - | - | - | - | - | - | - | - | - | - | - | - | - |
| **3. OI** | 0.001 | 0.35 | 0.003 | 0.32 | - | - | - | - | - | - | - | - | - | - | - | - | - | - |
| **4. EI** | NC | -0.22 | NC | -0.16 | NC | 0.07 | - | - | - | - | - | - | - | - | - | - | - | - |
| **5. Length** | < 0.001 | 0.62 | 0.001 | 0.36 | NC | 0.16 | 0.001 | -0.42 | - | - | - | - | - | - | - | - | - | - |
| **6. Width** | NC | 0.07 | NC | -0.14 | NC | 0.06 | NC | 0.27 | NC | 0.04 | - | - | - | - | - | - | - | - |
| **7. Thickness** | < 0.001 | 0.61 | 0.001 | 0.41 | NC | 0.05 | NC | -0.18 | < 0.001 | 0.67 | NC | 0.24 | - | - | - | - | - | - |
| **8. Weight** | < 0.001 | 0.55 | 0.004 | 0.32 | NC | 0.09 | NC | -0.28 | < 0.001 | 0.83 | 0.001 | 0.40 | < 0.001 | 0.81 | - | - | - | - |
| **9. Nº Scars** | < 0.001 | 0.88 | 0.001 | 0.65 | 0.003 | 0.32 | NC | -0.27 | < 0.001 | 0.68 | NC | 0.05 | < 0.001 | 0.54 | < 0.001 | 0.60 | - | - |

**Table E. Pearson Correlation Matrix among RII, OI, EI, length, width, thickness, weight, and number (nº) of scars.** Statically significant correlations were observed in accordance with Bonferroni correction and were only determined if p = ≤ 0.006 (p = p-value; r = correlation coefficient; NC = no correlation or p = >0.006).

| **Variable** | **1** | | **2** | | **3** | |
| --- | --- | --- | --- | --- | --- | --- |
|  | **p** | **r** | **p** | **r** | **p** | **r** |
| **1. RII** | - | - | - | - | - | - |
| **2. Length-edge** | 0.002 | 0.30 | - | - | - | - |
| **3. Angle-edge** | < 0.001 | 0.53 | < 0.001 | 0.34 | - | - |

**Table F.** **Pearson Correlation Matrix among RII, length-edge, and angle-edge**. Statically significant correlations were observed in accordance with Bonferroni correction and were only determined if p = ≤ 0.025 (p = p-value; r = correlation coefficient).

| **Comparison** | **Observed Difference** | **Critical Difference** |
| --- | --- | --- |
| **Unshaped-Unifacial** | 14.79 | 18.85 |
| **Unshaped-Bifacial** | 56.88 | 17.77 |
| **Unifacial-Bifacial** | 42.09 | 15.81 |

**Table G. Post-hoc test between RII and shaping**. Pairwise comparisons for the mean ranks of RII by levels of shaping.

| **Shaping** | **Delineation** | | | | |
| --- | --- | --- | --- | --- | --- |
|  | **Straight** | **Irregular** | **Pointed** | **Convex** | **Concave** |
| **Unshaped** | 4 [1.35] | 6 [2.02] | 8 [4.49] | 2 [12.57] | 2 [1.57] |
| **Unifacial** | 1 [1.96] | 2 [2.94] | 11 [6.53] | 13 [18.29] | 5 [2.29] |
| **Bifacial** | 1 [2.69] | 1 [4.04] | 1 [8.98] | 41 [25.14] | 0 [3.14] |

**Table H.** **Chi-square test between shaping and delineation**. Observed and expected frequencies by shaping and delineation. Items in brackets represent expected cell frequencies.

|  | **Nº Obj** | **Nº Schemes** | **Weight** | | | | **Reduction Intensity** | | | |
| --- | --- | --- | --- | --- | --- | --- | --- | --- | --- | --- |
|  |  |  | **Minim.** | **Maxim.** | **Mean** | **Std. D.** | **RI 0** | **RI 1** | **RI 2** | **RI 3** |
| **Vertical TFD** | | | | | | | | | | |
| **Tp1** | 11 | 2 | 529 | 3914 | 1385,5 | 939,13 | - | 4 | 5 | 2 |
| **Tp3** | 15 | 2 | 380 | 3660 | 920,9 | 712,82 | 4 | 8 | 1 | 2 |
| **Horizontal TFD** | | | | | | | | | | |
| **Tp3** | 18 | 2 | 232 | 2730 | 609,9 | 474,6 | 9 | 7 | 1 | 1 |
| **Tp2** | 2 | 1 | 337 | 685 | 506 | 179 | - | 2 | - | - |
| **Combined TFD** | | | | | | | | | | |
| **Tp3** | 9 | 3 | 232 | 2738 | 989,2 | 723,24 | 4 | 1 | 2 | 2 |
| **Tp2** | 29 | 7 | 183 | 2350 | 806,8 | 525,01 | 10 | 10 | 8 | 1 |

**Table I. Tool functioning direction (TFD).** Number of objects, tool functioning schemes, weight and types of reduction intensity (RI) sorted by type of TFD and artifact type (Tp) (Minim. = minimum; maxim. = maximum; Std. D. = standard deviation).

| **TFD** | **Tp1** | **Tp2** | **Tp3** |
| --- | --- | --- | --- |
| **Vertical** | 10 [3.57] | 15 [15.36] | 5 [11.07] |
| **Horizontal** | 0 [3.93] | 9 [16.89] | 24 [12.18] |
| **Combined** | 0 [2.50] | 19 [10.75] | 2 [7.75] |

**Table J.** **Chi-square test between tool functioning direction (TFD) and tool-type.** Observed and expected frequencies by TFD and tool-type. Items in brackets represent expected cell frequencies.

| **TFD** | **Geometry** | |
| --- | --- | --- |
|  | **Dihedral** | **Trihedral** |
| **Vertical** | 12 [8.21] | 1 [4.79] |
| **Horizontal** | 42 [27.79] | 2 [16.21] |
| **Combined** | 18 [36.00] | 39 [21.00] |

**Table K.** **Chi-square test between tool functioning direction (TFD) and edge geometry**. Observed and expected frequencies by TFD and geometry. Items in brackets represent expected cell frequencies.

| **Comparison** | **Observed Difference** | **Critical Difference** |
| --- | --- | --- |
| **Vertical-Horizontal** | 13.76 | 16.30 |
| **Vertical-Combined** | 5.18 | 14.73 |
| **Horizontal-Combined** | 18.93 | 16.61 |

**Table L.** **Post-hoc test between RII and tool functioning direction (TFD)**. Pairwise comparisons for the mean ranks of RII by levels of TFD.

| **Comparison** | **Observed Difference** | **Critical Difference** |
| --- | --- | --- |
| **Vertical-Horizontal** | 10.80 | 16.30 |
| **Vertical-Combined** | 25.95 | 14.73 |
| **Horizontal-Combined** | 15.15 | 16.61 |

**Table M.** **Post-hoc test between OI and tool functioning direction (TFD)**. Pairwise comparisons for the mean ranks of OI by levels of TFD.

| **Comparison** | **Observed Difference** | **Critical Difference** |
| --- | --- | --- |
| **Vertical-Horizontal** | 11.50 | 16.21 |
| **Vertical-Combined** | 7.66 | 14.66 |
| **Horizontal-Combined** | 19.16 | 16.42 |

**Table N.** **Post-hoc test between EI and tool functioning direction (TFD)**. Pairwise comparisons for the mean ranks of EI by levels of TFD.

| **Comparison** | **Observed Difference** | **Critical Difference** |
| --- | --- | --- |
| **Vertical-Horizontal** | 18.33 | 16.30 |
| **Vertical-Combined** | 3.71 | 14.73 |
| **Horizontal-Combined** | 22.04 | 16.61 |

**Table O. Post-hoc test between weight and tool functioning direction (TFD)**. Pairwise comparisons for the mean ranks of weight by levels of TFD.

| **TFD** | **Weight group 1** | **Weight group 2** |
| --- | --- | --- |
| **Vertical** | 27 [21.43] | 3 [8.57] |
| **Horizontal** | 25 [23.57] | 8 [9.43] |
| **Combined** | 8 [15.00] | 13 [6.00] |

**Table P.** **Chi-square test between tool functioning direction (TFD) and weight group**. Observed and expected frequencies by TFD and weight group.

| **Action** | **TFD** | | |
| --- | --- | --- | --- |
|  | **Vertical** | **Horizontal** | **Combined** |
| **Resting** | 6 [8.25] | 12 [5.25] | 3 [7.50] |
| **Combined** | 8 [8.25] | 3 [5.25] | 10 [7.50] |
| **Thrusting** | 19 [16.50] | 6 [10.50] | 17 [15.00] |

**Table Q.** **Chi-square test between action and tool functioning direction (TFD)**. Observed and expected frequencies by action and TFD. Items in brackets represent expected cell frequencies.

| **Action** | **Blank** | | | |
| --- | --- | --- | --- | --- |
|  | **Flake** | **Block** | **Slab** | **Splitted** |
| **Resting** | 20 [12.25] | 1 [2.75] | 0 [3.25] | 0 [2.75] |
| **Combined** | 15 [12.25] | 1 [2.75] | 3 [3.25] | 2 [2.75] |
| **Thrusting** | 14 [24.50] | 9 [5.50] | 10 [6.50] | 9 [5.50] |

**Table R. Chi-square test between action and blank**. Observed and expected frequencies by action and blank. Items in brackets represent expected cell frequencies.

| **Comparison** | **Observed Difference** | **Critical Difference** |
| --- | --- | --- |
| **Resting-Combined** | 21.00 | 18.02 |
| **Resting-Thrusting** | 52.50 | 15.61 |
| **Combined-Thrusting** | 31.50 | 15.61 |

**Table S. Post-hoc test between weight and action**. Pairwise comparisons for the mean of weight by levels of action.

| **Comparison** | **Observed Difference** | **Critical Difference** |
| --- | --- | --- |
| **Resting-Combined** | 9.38 | 18.02 |
| **Resting-Thrusting** | 25.79 | 15.61 |
| **Combined-Thrusting** | 16.40 | 15.61 |

**Table T. Post-hoc test between RII and action**. Pairwise comparisons for the mean ranks of RII by levels of action.

| **TFD** | **Delineation** | | | |
| --- | --- | --- | --- | --- |
|  | **Straight** | **Irregular** | **Pointed** | **Convex** |
| **Horizontal** | 40 [40.53] | 18 [14.00] | 1 [4.42] | 11 [11.05] |
| **Vertical** | 15 [14.47] | 1 [5.00] | 5 [1.58] | 4 [3.95] |

**Table U. Chi-square test between tool functioning direction (TFD) and delineation**. Observed and expected frequencies by TFD and delineation. Items in brackets represent expected cell frequencies.

| **Comparison** | **Observed Difference** | **Critical Difference** |
| --- | --- | --- |
| **Resting-Combined** | 7.47 | 18.43 |
| **Resting-Thrusting** | 18.56 | 15.86 |
| **Combined-Thrusting** | 11.09 | 17.05 |

**Table W. Post-hoc test between length-edge and action**. Pairwise comparisons for the mean ranks of length-edge by levels of action.

**Fig A**. **Mean rank of RII by blank**. Boxplots of the ranked values of RII by the levels of blank.

**Fig B.** **Correlation between pairs of variables of tool shape and size**. Scatterplot matrix among RII, SDI, OI, EI, length, width, thickness, weight, and number (nº) of scars.

**Fig C*.* Mean rank of RII by tool-type.** Boxplots of the ranked values of RII by the levels of tool-type.

**Fig D.** **Mean rank of SDI by tool-type**. Boxplots of the ranked values of SDI by the levels of tool-type.

**Fig E.** **Mean rank of OI by tool-type**. Boxplots of the ranked values of OI by the levels of tool-type.

**Fig F.** **Mean rank of EI by tool-type**. Boxplots of the ranked values of EI by the levels of tool-type.

**Fig G. Mean rank of length by tool-type.** Boxplots of the ranked values of length by the levels of tool-type.

**Fig H. Mean rank of thickness by tool-type**. Boxplots of the ranked values of thickness by the levels of tool-type.

**Fig I. Mean rank of weight by tool-type**. Boxplots of the ranked values of weight by the levels of tool-type.

**Fig J. Mean rank of nº of scars by tool-type**. Boxplots of the ranked values of number (nº) of scars by the levels of tool-type.

**Fig K** **Correlation between pairs of variables of RII and cutting-edge variables**. Scatterplot matrix among RII, length-edge, and angle-edge.

**Fig L. Mean rank of RII by shaping** **type**. Boxplots of the ranked values of RII by the levels of shaping.

**Fig M. Mean rank of RII by tool functioning direction (TFD)**. Boxplots of the ranked values of RII by the levels of TFD.

**Fig N. Mean rank of OI by tool functioning direction (TFD)**. Boxplots of the ranked values of OI by the levels of TFD.

**Fig O. Mean rank of EI by tool functioning direction (TFD)**. Boxplots of the ranked values of EI by the levels of TFD.

**Fig P. Mean rank of weight by tool functioning direction (TFD)**. Boxplots of the ranked values of weight by the levels of TFD.

**Fig Q.** **Mean rank of weight by action**. Boxplots of the ranked values of weight by the levels of action.

**Fig R*.* Mean rank of RII by action**. Boxplots of the ranked values of RII by the levels of action.

**Fig S. Mean rank of length-edge by tool functioning direction (TFD)**. Boxplots of the ranked values of length by the levels of TFD.

**Fig T*.* Mean rank of length-edge by action.** Boxplots of the ranked values of length by the levels of action.
